# Bilateral retinal artery occlusion as the initial presentation of infectious endocarditis: a case report

**DOI:** 10.1186/s13256-025-05141-1

**Published:** 2025-03-14

**Authors:** Ana Bárbara Dias Lopes Urzedo, Bruna Predabon, Márcio Adriano Filho, Germano Ramos Boff, Kenzo Hokazono

**Affiliations:** Department of Ophthalmology, Clinical Hospital of the Federal University of Paraná, R. da Paz, 195 (123), Curitiba, PR 80060-160 Brazil

**Keywords:** Central retinal artery occlusion, Infectious endocarditis, *Erysipelothrix rhusiopathiae*

## Abstract

**Background:**

Retinal artery occlusion due to infective endocarditis is rare, and only a few cases have been reported in literature. Systematic and comprehensive studies of patients with this ophthalmological disease may help uncover serious underlying medical conditions.

**Case presentation:**

We present a case of an 80-year-old Brazilian woman with bilateral retinal artery occlusion as the initial presentation of *Erysipelothrix rhusiopathiae* endocarditis.

**Conclusion:**

Infective endocarditis has many different forms of presentation and a high clinical suspicion is often required to reach a diagnosis. This case report highlights the importance of remembering retinal artery occlusion as a complication of infective endocarditis even if concurrent cardiovascular risk factors are present.

## Introduction

Central retinal artery occlusion (CRAO) is a rare but devastating disorder that typically presents as sudden painless loss of vision in the involved eye. The obstruction of blood flow to the central retinal artery (CRA) has several etiologies, but the most common etiology is embolism from an atherosclerotic plaque from the internal carotid artery [1, 2]. Retinal artery occlusion (RAO) secondary to infectious endocarditis (IE) is a rare event with only a few cases reported in literature. The authors report the first case of bilateral RAO due to *Erysipelothrix rhusiopathiae* endocarditis. Given the wide range of clinical presentations of IE, this case report highlights the importance of remembering RAO as a complication of IE, since accurate diagnosis at early stages is essential, otherwise severe complications including heart failure may occur.

## Case presentation

An 80-year-old Brazilian woman was referred to the ophthalmology department with sudden and painless vision loss in the right eye (OD) for 3 days followed by her left eye (OS) upon awakening on the morning of admission. She had a 10-day history of malaise, episodes of sweating, and unmeasured fever. Her medical history included carotid atherosclerotic disease, ischemic heart failure with preserved ejection fraction, systemic arterial hypertension, insulin-dependent diabetes mellitus, and dyslipidemia.

**Fig. 1:**
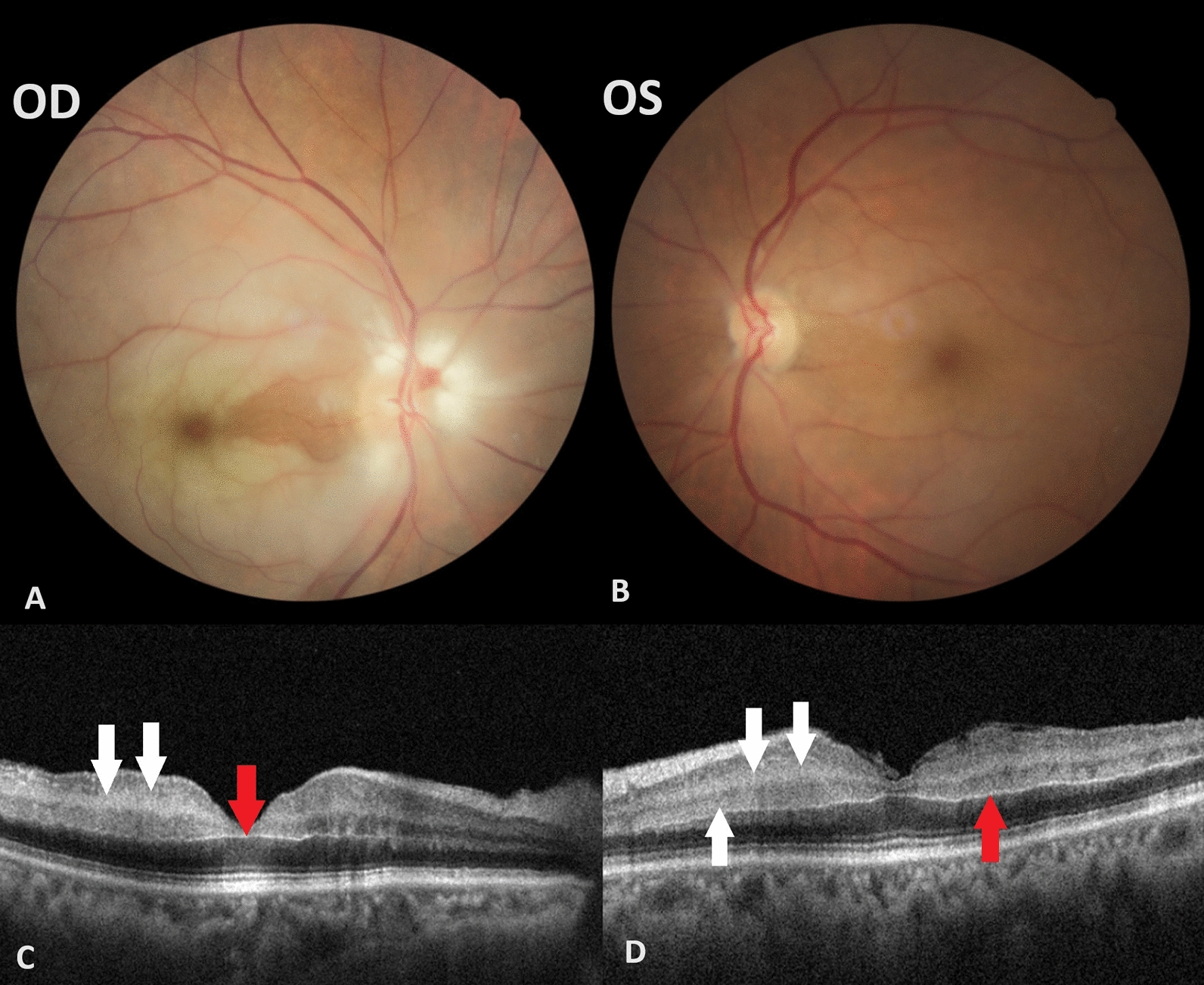
Fundus photography of the right eye showing marked ischemic retinal whitening with a “cherry red spot” sign at the macula with cilioretinal artery sparing and narrow arterioles. **A** Fundus image of the left eye showing slight retinal pallor in the nasal fovea and a narrowed arteriole in the papillomacular bundle. **B** Optical coherence tomography B-scan of the right eye (**C**) and left eye (**D**) demonstrating thickening and hyperreflectivity of the inner nuclear layers (white arrows), sparing the outer retina, and a prominent middle limiting membrane (red arrow), which are typical signs of acute ischemia

On examination, best-corrected visual acuity (BCVA) was counting fingers OD and 0.2 OS, biomicroscopy was unremarkable, except for pseudophakia, and intraocular pressure (IOP) was 9 mmHg bilaterally. Dilated fundoscopy revealed diffuse whitening of the retina except in the cilioretinal artery territory, a “cherry red spot” on the macula and narrow arterioles in the OD. In OS, there was a slight retinal pallor in the nasal fovea and a narrowed arteriole in the papillomacular bundle (Fig. 1).

Immediately after the diagnosis of bilateral retinal arterial occlusion, the patient was referred for systemic evaluation. Cardiac auscultation revealed a holosystolic murmur in the mitral focus. Laboratory tests revealed normocytic normochromic anemia and rheumatoid factor positive, while C-reactive protein levels and the erythrocyte sedimentation rate were slightly increased. Doppler ultrasound of the temporal arteries was negative for giant cell arteritis owing to the absence of the halo sign. Transesophageal echocardiography (TEE) and blood culture were performed. TEE revealed mobile vegetation approximately 14 mm in size adhering to the atrial surface of the mitral leaflet, and blood cultures were positive six times, with *Erysipelothrix rhusiopathiae*. Magnetic resonance imaging (MRI) revealed some acute cerebral ischemic foci.

The patient was treated with intravenous ampicillin for 6 weeks, with venous thromboembolism prophylaxis, surveillance for immunological events, and serial blood cultures for control. Owing to the absence of signs of acute heart failure, such as hemodynamic instability or acute pulmonary edema, or signs of local infectious complications, such as abscesses, fistulas or growing vegetation, cardiac surgical treatment was not recommended.

The patient was discharged after full clinical stabilization 51 days after admission. At the time of the last ophthalmologic examination, the BCVA OD was 0.05, and OS was 0.5, and fundoscopy of both eyes remained unchanged.

## Discussion

CRAO is a rare but devastating disorder that typically presents as sudden painless loss of vision in the involved eye. The incidence of CRAO is approximately 1–2 in 100,000, but may be higher in patients older than 80 years. The obstruction of blood flow to the CRA has several etiologies, but the most common is embolism from an atherosclerotic plaque from the internal carotid artery and, because of that, CRAO risk factors are similar to those for cardiovascular and cerebrovascular events, such as hypertension and diabetes mellitus [1, 2].  RAO secondary to IE is a rare event with only a few cases reported in literature.

IE is a highly morbid disease with an incidence ranging from 1.4 to 6.2 per 100,000 [3]. Several risk factors for the development of IE have been described, including structural heart disease, prosthetic heart valves, injection drug use, and a history of IE [4]. Systemic embolization occurs in 22–50% of cases and the risk seems to be greater with mitral valve involvement and a large vegetation size, as our patient presented [5]. These embolus commonly affect regions with large vessels, such as the lungs and coronary arteries. However, 65% of embolic events affect the central nervous system, especially the territory of the middle cerebral artery [6]. Therefore, these data are consistent with the fact that CRAO due to an embolus from cardiac vegetation is a very rare complication of IE, occurring in less than 1% of cases [7].

IE on native valves is most commonly caused by *Streptococcus viridans* and *Staphylococcus*. *Erysipelothrix rhusiopathiae* is a facultative anaerobic Gram-positive bacillus that causes erysipelas-like syndrome in wild and domestic animals as a zoonotic pathogen. Human infection is rare, but three forms of *E*. *rhusiopathiae* infection have been described: local cutaneous disease (most common), generalized cutaneous infection, and septicemia, which are commonly associated with endocarditis and a high mortality rate [8]. In a systematic review of 62 cases, 23 patients (37.1%) had cardiac valve involvement, 14 of whom underwent valve replacement surgery. Thus, accurate diagnosis at early stages is essential; otherwise, severe complications, including heart failure, may occur [9].

Our patient had several cardiovascular risk factors and the CRAO could have been attributed to this, if we had not considered her systemic complaints that pointed toward an inflammatory/infectious disease. Furthermore, given the presence of bilateral RAO with systemic symptoms, vasculitis, such as temporal arteritis, should be included in the differential diagnosis, as in this case, early steroid treatment could spare the patient’s eye.

## Conclusion

Given the wide range of clinical presentations of IE, this case report highlights the importance of remembering RAO as a complication of IE even if concurrent cardiovascular risk factors are present. To the best of our knowledge, this is the first case report of bilateral RAO leading to the diagnosis of IE, a life-threatening disease.

## Data Availability

Data are available within the article or its supplementary materials.
